# 2615. Effect of Early NSAID and Aspirin Use on Clinical Outcomes in Patients Hospitalized with Influenza: A Retrospective Study

**DOI:** 10.1093/ofid/ofad500.2228

**Published:** 2023-11-27

**Authors:** Suk Yin Chan-Colenbrander, Qi Wang

**Affiliations:** University of Minnesota, Minneapolis, Minnesota; University of Minnesota, Minneapolis, Minnesota

## Abstract

**Background:**

Seasonal influenza infection causes significant morbidity and mortality worldwide. This study evaluates the effect of early nonsteroidal anti-inflammatory drug (NSAID) and aspirin use in patients hospitalized with influenza.

**Methods:**

We performed a retrospective chart review of patients (age 18 years and older) admitted between January 1, 2016, and December 31, 2019, to the University of Minnesota Medical Center, who were tested positive for influenza. Data were summarized using median and interquartile range (IQR) for continuous variables, frequency and percentage for categorical variables. Groups were compared using two-sample Wilcoxon test for continuous variables and Chi-square test (or Fisher's exact test for sparse data) for categorical variables.

**Results:**

Out of 320 patients tested positive for influenza, 180 were vaccinated against influenza. The median age of those tested positive and vaccinated was 64 (IQR 49-74) and those unvaccinated, 58 (IQR 35-71), p=0.0074. 97.8% of the vaccinated and 94.3% of the unvaccinated, received oseltamivir. More unvaccinated patients were admitted to the intensive care unit (ICU) (p=0.0029) and required ventilatory support (p=0.0097) compared to those vaccinated.

Among the vaccinated, early NSAID use was associated with fewer cardiovascular complications, but this was not significant (p=0.0758). NSAID use was also associated with a higher chance of survival at 1 year (p=0.0004) and 3 years (p< 0.0001) post hospitalization. Among the unvaccinated, NSAID use led to fewer ICU admissions (p=0.0401), fewer cardiovascular complications (p=0.0156) and fewer renal complications (p=0.0006).

Those who were vaccinated and not on aspirin, had a better chance of surviving the hospital stay compared to those on aspirin (p=0.0452). Those who were unvaccinated and not on aspirin, had a higher chance of survival at 3 years post hospitalization than those on aspirin (p=0.0131). No significant difference was observed with aspirin use between these 2 groups in cardiovascular, respiratory or renal complications, ICU admission, 30 days readmission or survival at 1 year post hospitalization.

**Conclusion:**

Our findings show better outcomes with early NSAID use but worse outcomes with aspirin in patients hospitalized with influenza

**Disclosures:**

**All Authors**: No reported disclosures

